# Enzyme-treated chicory for cosmetics: application assessment and techno-economic analysis

**DOI:** 10.1186/s13568-022-01494-8

**Published:** 2022-12-06

**Authors:** Suvi Tuulikki Häkkinen, Katarina Cankar, Liisa Nohynek, Marjut Suomalainen, Jeroen van Arkel, Matti Siika-Aho, Anna Twarogowska, Bart Van Droogenbroeck, Kirsi-Marja Oksman-Caldentey

**Affiliations:** 1 VTT Technical Research Centre of Finland Ltd, Tietotie 2, 02044 Espoo, Finland; 2 Wageningen Plant Research, Wageningen University & Research, Wageningen, The Netherlands; 3 ILVO Institute for Agriculture, Fisheries and Food Research, Melle, Belgium

**Keywords:** Chicory, By-product, Antimicrobial activity, Enzyme treatment, Techno-economic analysis

## Abstract

Chicory (*Cichorium intybus* L.) is an important industrial crop that produces large quantities of the dietary fiber inulin in its roots. Following inulin extraction, the bagasse is typically used as animal feed, but it contains numerous bioactive secondary metabolites with potential applications in healthcare and cosmetic products. Here we assessed the antimicrobial properties of chicory biomass pre-treated with various enzymes alone and in combination to release the bioactive compounds and increase their bioavailability. We found that pre-treatment significantly increased the antimicrobial activity of this industrial by-product, yielding an extract that inhibited typical skin pathogens in a cosmetic formula challenge test. We also evaluated the valorization of chicory biomass as a bioactive cosmetic ingredient. Economic feasibility was estimated by combining our experimental results with a conceptual techno-economic analysis. Our results suggest that chicory biomass can be utilized for the sustainable production of efficacious cosmetic ingredients.

**Supplementary Information:**

The online version contains supplementary material available at 10.1186/s13568-022-01494-8.

## Keypoints

- Industrial chicory waste streams can be upgraded.
- Chicory by-product was treated with enzymes to produce an antimicrobial ingredient.
- Process is profitable and product suitable for cosmetic/personal care applications.

**Supplementary Information:**

The online version contains supplementary material available at 10.1186/s13568-022-01494-8.

## Introduction

Chicory (*Cichorium intybus* L.) is a perennial woody herb (Asteraceae) whose root tissue consists of up to 40% of the dietary fiber inulin (HMPC 2013). There are four main varieties or cultigroups defined according to their use (Street et al. 2013): (1) industrial or root chicory, whose taproot is used as a coffee substitute or for inulin extraction; (2) Belgian endive or witloof chicory, which is cultivated for its etiolated buds, known as chicons; (3) leaf chicory or radicchio, which is used in salads or as cooked vegetables; and (4) forage chicory, initially derived from wild chicory, which is used to feed livestock. The major variety is industrial chicory, 1.8 million tons of which is cultivated annually in Europe (van Arkel et al. 2012) (Endive Biennale 2016).

The major product extracted from chicory is inulin, but it also produces many bioactive secondary metabolites that could enhance its value. These include sesquiterpenes, polyphenols and other phenolic compounds and their derivatives (Street et al. 2013; Bogdanović et al. 2019). Chicory sesquiterpenes were previously found to possess anti-inflammatory, antimicrobial and sedative activities (Häkkinen et al. 2021; Matos et al. 2020; Wesołowska et al. 2006). Antimicrobial activity has been confirmed against *S. aureus*, *Bacillus* spp. and *E. coli* (Liu et al. 2013), as well as *A. tumefaciens*, *P. carotovorum*, *P. fluorescens* and *P. aeruginosa* (Petrovic et al. 2004).

Antimicrobial ingredients are included in cosmetic products as preservatives and to stabilize the skin microbiome. Synthetic preservatives such as triclosan, parabens and methylisothiazolinone are no longer allowed in cosmetic products, increasing the demand for natural alternatives in a global cosmetic preservative market that is expected to reach US$ 458.8 million by 2024 (Frost and Sullivan 2019). Accordingly, the revenue of natural preservatives in cosmetics will reach US$ 140.5 million in 2023 and further growth is predicted (Frost and Sullivan 2019). One important challenge is the extraction of such bioactive ingredients to increase their efficacy. Pectinolytic enzymes are used in the juice processing industry to facilitate extraction, and most commercial pectinase preparations are mixtures of endoacting (carbohydrate backbone cleaving) and exoacting (carbohydrate side chain cleaving) pectinases together with cellulases and hemicellulases. Enzymatic treatment enhances the extraction of phenolic compounds from plant cell walls (Landbo and Meyer 2001). The exoacting properties of these enzymes can also directly influence the chemistry and therefore the bioactivity of phenolic glycosides (Buchert et al. 2005; Puupponen-Pimiä et al. 2008).

Here we investigated the potential of chicory bagasse, the industrial by-product remaining after inulin extraction, as a source of bioactive secondary metabolites for cosmetic products supporting the human skin welfare. We tested various enzymes alone and in combination in order to enhance the antimicrobial activity of extracts in cosmetic formula challenge tests. In addition to human skin pathogens *S. aureus* and *P. aeruginosa*, the antimicrobial effect of the extracts was evaluated with human probiotic bacteria *L. rhamnosus* and *S. thermophilus*. We also carried out a techno-economic analysis of the production process to determine whether chicory biomass can be profitably upgraded into sustainable and effective cosmetic ingredients.

## Materials and methods

### Chicory material

Forced roots of Belgian endive (*Cichorium intybus* var. *foliosum*) (sample #1) were provided by Flanders Research Institute for Agriculture, Fisheries and Food (ILVO). Industrial chicory roots (*Cichorium intybus* var. *sativum*), before (sample #2) and after (sample #3) inulin extraction, were provided by CoSucra Groupe Warcoing (Warcoing, Belgium). Unprocessed chicory roots (sample #2) were washed and cut into cossettes before inulin extraction using a countercurrent hot water extractor. This produced raw juice containing inulin and exhausted chicory cossettes, which were pressed to yield bagasse containing ~ 25% dry matter (sample #3). All samples were dried in a hot air oven at 60 °C for 6–8 h until the moisture content fell below 10%. Dried samples were processed in a ZM 200 ultracentrifugal mill fitted with a 0.5-mm sieve mesh (Retsch, Haan, Germany). The composition of each sample is summarized in Additional file 1: Table S1.

### Enzymatic treatment

Samples were suspended in 0.05 M acetate buffer (pH 5.0) to dry matter content of 5% (w/v) with a total volume of 20 mL, and were mixed at 50 °C for 24 h in an HT Minitron temperature cabinet (Infors, Bottmingen-Basel, Switzerland). We used six enzymes alone or in combinations: inulinase (Fructozyme L), pectinase (Pectinex Smash), cellulase (Cellic CTec2) and β-glucosidase (Novozym 188), all from Novozymes (Bagsværd, Denmark), as well as xylanase (Depol 40L) and ferulic acid esterase (Depol 740L) from Biocatalysts (Cardiff, UK). The enzyme dose was calculated based on the protein content. At the end of each treatment, the enzymes were inactivated by boiling the sample for 15 min, followed by centrifugation (2600 ×*g*, 15 min, 23 °C). The supernatants were stored at − 20 °C.

### Quantification of reducing sugars

The sugars released by enzymatic hydrolysis were analyzed using a reducing sugar assay based on the dinitrosalicylic acid (DNS) colorimetric method in a 96-well format (Silveira et al. 2014).

### Antimicrobial activity assay

Antimicrobial activity was measured using the liquid culture method in a total volume of 1 mL (Puupponen-Pimiä et al. 2016). We screened the following microbial strains: *Staphylococcus aureus* VTT E-70045 (ATCC 6538), *Pseudomonas aeruginosa* VTT E-84219 (ATCC 15692), *Lactobacillus rhamnosus* GG VTT E-96666 (ATCC 53103) and *Streptococcus thermophilus* VTT E-96665 (ATCC 19258). *S. aureus* and *P. aeruginosa* were cultivated aerobically in BD Difco nutrient broth (Thermo Fisher Scientific, Waltham, MA, USA) at 37 °C, shaking at 150 rpm. *L. rhamnosus* and *S. thermophilus* were cultivated in De Man Rogosa Sharpe (MRS) broth (Oxoid, Basingstoke, UK) at 37 °C without agitation. Microbial stock cultures were stored at − 80 °C. Before cultivation, they were grown on solid media for 1–2 days as described above for each strain. Single colonies were transferred to liquid media, incubated for 20–24 h, and used as the source of inoculum for antimicrobial activity tests. Microbial cultures incubated with enzyme(s) in 100 µL acetic acid buffer without antimicrobial samples were used as positive growth controls. Cultures were incubated with 50 µg/mL chloramphenicol in 100 µL acetic acid buffer as a negative control. The inhibitory effects of chicory samples suspended in 100 µL acetic acid buffer were evaluated by comparison with the control growth curves.

### Sugar analysis

Sugars were extracted as previously described (Muir et al. 2009) with minor changes. Briefly, 100 mg of powder was mixed with 8 mL distilled water in a 10-mL volumetric flask by sonication for 15 min at 80 °C in a Branson 3510 device (Marshal Scientific, Hampton, NH, USA). After cooling to room temperature (25 °C), the volume was adjusted to 10 mL with distilled water and the sample was centrifuged (3000 ×*g*, 10 min, room temperature). The supernatant was passed through a 0.22-µm sterile Millex filter (Merck, Overijse, Belgium) and analyzed by size exclusion chromatography (SEC) using an Acquity H-Class ultra-high performance liquid chromatography (UPLC) system (Waters, Milford, MA, USA) with a refractive index detector and external column oven. We used two TSK gel G2500PWXL columns (Tosoh, Tokyo, Japan) at 80 °C and a guard column (Bio-Rad, Temse, Belgium) under isocratic conditions, with distilled water as the mobile phase at a flow rate of 0.5 mL/min. The injected volume was 10 µL, and each run lasted 40 min. Sugars were identified by retention time and quantified against calibration curves of sucrose, fructose, glucose, raffinose and stachyose standards (Merck).

### Sesquiterpene lactones

Sesquiterpene lactones (STLs) were extracted as previously described (Kips, 2017) with minor changes. Briefly, we added 74 µL of the internal standard (10 µg/mL santonin) to 50 mg of powdered sample, followed by extraction with 1.405 mL deionized water containing 0.1% formic acid. The sample was incubated for 15 min at 30 °C, shaking at 1300 rpm on a thermomix comfort (Eppendorf, Rotselaar, Belgium). It was then centrifuged (20,817 × *g*, 15 min, room temperature), and the supernatant was passed through a 0.22-μm Millex filter. STLs were separated on the Waters Acquity UPLC system using a BEH C18 column (150 mm × 2.1 mm, 1.7 μm). The mobile phase was a mixture of deionized water plus 0.1% formic acid (solvent A) and acetonitrile plus 0.1% formic acid (solvent B) at a flow rate of 0.350 mL/min. The gradient started at 5% B for 5 min, followed by a linear increase from 5 to 53% B in 20 min, a hold for 1 min, with a final phase at 100% solvent B for 3 min. The column was then re-equilibrated to 5% B for 4 min before the next injection. The column temperature was 40 °C and the injection volume was 5 µL. STLs were detected by high-resolution mass spectrometry (HRMS) using a Waters Synapt G2-S quadrupole time-of-flight (QTOF) instrument in positive electrospray ionization (ESI +) MSE mode. Before sample analysis, the HRMS was calibrated (50–1200 Da) using sodium formate solution. The HRMS was operated in resolution mode (20,000 FWHM) and a leucine-enkephalin solution (200 pg/µL) was constantly infused during analysis as lockmass.

Four compounds were quantified against reference standards (Extrasynthese, Genay, France): lactucin, lactucopicrin, dihydrolactucin and dihydrolactucopicrin. For oxalates and glycosides, no standards are commercially available so we based our analysis on relative peak areas. Data were recorded using MassLynx v4.1 and integrated using TargetLynx v4.1 (Waters).

### Phenolic compounds

Phenolic compounds were extracted as previously described (Kips 2017). Briefly, 75 mg of sample powder was mixed with the internal standard (1 μg/g daidzein) and extracted with 5 mL 100% methanol followed by 5 mL 20:80 (v/v) methanol:water. Each extraction step consisted of 1 min vortexing and 15 min ultrasound-assisted extraction using a Transsonic Digital S device (Elma Schmidbauer, Singen, Germany), followed by centrifugation (3000 ×*g*, 15 min, room temperature). The supernatants were combined and passed through a 0.22-μm Millex filter before separation on the Waters Acquity UPLC system fitted with a BEH C18 column (150 mm × 2.1 mm, 1.7 μm). The mobile phase was a mixture of water plus 0.1% formic acid (solvent A) and acetonitrile plus 0.1% formic acid (solvent B) at a flow rate of 196 µL/min. The gradient increased in a linear fashion from 1 to 24% B in 9.9 min, then to 65% B at 18.5 min and to 99% B at 18.7 min, followed by a hold at 99% B until 20.7 min. The column was then restored to 99% A from 20.8 to 23 min before the next injection. The column temperature was kept at 40 °C and the injection volume was 5 µL. Fractions were analyzed using a Xevo TQ-S tandem mass spectrometer (Waters) in electrospray ionization negative (ESI-) mode with multiple reaction monitoring (MRM). Phenolic compounds were quantified based on relative peak areas and against the reference standards chlorogenic acid, chicoric acid, quinic acid, caffeic acid and 4-OH-phenylacetic acid (Merck). Data were recorded and integrated as described above.

Phenolic compounds were also evaluated by non-targeted Fourier transform mass spectrometry (FTMS) as previously described (Cankar et al. 2021). Briefly, 300-µL samples were extracted with 700 µL methanol containing 0.1% formic acid by vortexing and sonication as above, followed by centrifugation (21,000 ×*g*, 15 min, room temperature). The supernatants were then fractionated on an LC-PDA-LTQ-Orbitrap-FTMS system (Thermo Fisher Scientific) comprising an Acquity H-Class UPLC fitted with an Acquity elambda photodiode array (PDA) detector (220–600 nm) connected to an LTQ/Orbitrap XL hybrid mass spectrometer with an ESI interface. Raw LC-Orbitrap-FTMS data were processed using MetAlign (Lommen 2009) to correct for background and noise and to align the chromatographic peaks from the different samples. Values lower than the detection threshold (s/n ≥ 3) were removed using MetAlign Output Transformer (Houshyani et al. 2012), and the remaining mass peaks were clustered into centrotypes using MSClust (Tikunov et al. 2012) based on original retention times and peak intensities across all samples. For sample #3A (chicory biomass treated with pectinase plus xylanase), the most differential mass peaks compared to the other treatments were selected using Pearson’s correlation with a cut-off of 0.98. To exclude the least abundant metabolites, mass peaks with a tenfold lower mass peak area than average area were omitted.

For gas chromatography mass spectrometry (GC–MS), 0.5-mL samples were extracted with 1 mL dichloromethane by vortexing and sonication as above, followed by centrifugation (240 ×*g*, 10 min, room temperature). The supernatant was dehydrated in sodium sulfate and analyzed on GC–MS 7890A device coupled to a 5975A mass-selective detector (Agilent, Santa Clara, CA, USA). Analytes from 1-μL samples were separated on a Phenomenex (Torrance, CA, USA) 30 m × 0.25 mm ZB-5 column (0.25 mm film thickness) using helium as the carrier gas at a flow rate of 1 mL/min. The injector was used in splitless mode with the inlet temperature set to 250 °C. The initial oven temperature of 45 °C was increased after 1 min to 300 °C at a rate of 10 °C/min and held for 5 min at 300 °C.

### Cosmetic formula challenge test

Extracts of chicory by-product samples #3A (chicory biomass treated with pectinase plus xylanase) and #3D (untreated biomass) were added to a cosmetic cream formula (Additional file 1: Table S2) and tested for antimicrobial activity against *S. aureus* (VTT E-70045, ATCC 6538) and *P. aeruginosa* (VTT E-96728, ATCC 9027) at cell densities of 10^5^–10^6^ cfu/g formula according to ISO 11930 (Preservative Effectiveness Test), with some modifications. Chicory extracts were added at a concentration of 4% (v/v). We transferred 10 g of the sterile formula to a 50-mL Falcon tube and added the chicory extracts, which were filter sterilized and diluted with an equal volume of sterile glycerol before mixing. As a positive control, we used the commercial preservative Euxyl PE 9010 (containing phenoxyethanol and ethylhexylglycerol) at a concentration of 0.5% in the formula. As a negative control, we used the cream formula without preservative or chicory extract.

Microbial cultures were incubated at 37 °C for 18–24 h on tryptone soy agar (TSA) and were subcultured once before the experiment. A loop of bacterial biomass was collected from the plate and transferred to 5 mL of peptone-saline solution in a 20-mL tube with glass beads, and cell aggregates were disrupted by vortexing. The density of the suspension was adjusted with peptone-saline to Mac Farland 0.5 using densitometer (corresponding to 10^7^–10^8^ cfu/mL). Microbial inoculum (100 µL) was then added to the formula by vortexing and incubating at 25 °C. Samples (0.5-g) were collected from each culture (including controls) weekly for 4 weeks, resuspended in 4.5 mL peptone-saline and serially diluted before plating on TSA. The plates were incubated at 37 °C and colonies were counted after 24, 48 and 72 h.

### Techno-economic analysis

**Fig. 1:**
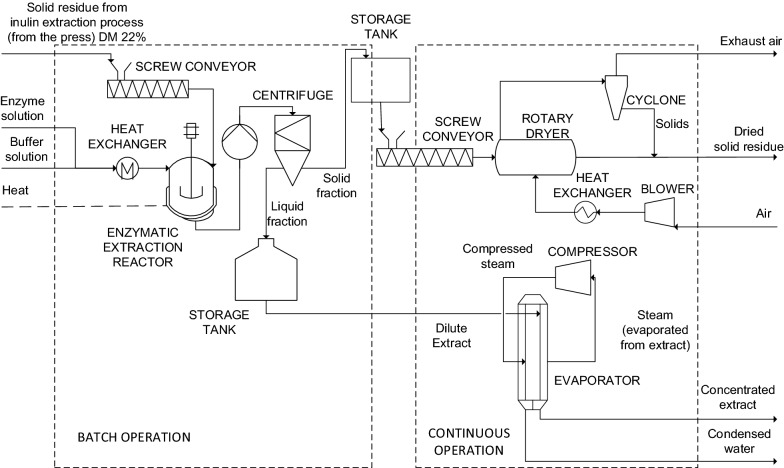
Baseline process (scenario 1) for the production of antimicrobial extracts from chicory biomass following inulin extraction

A conceptual level techno-economic analysis was carried out using sample #3 (Additional file 1: Table S1). In the baseline case (scenario 1), we processed 1500 tons of dry pulp with a dry matter (DM) content of 90%. The process is shown in Fig. 1. We also considered three alternative scenarios (Additional file 1: Table S3) in which the extract concentration step was omitted (scenario 2), fresh pulp was used instead of dry pulp (scenario 3), and the treatment was integrated with an inulin plant (scenario 4).

The process operating and performance parameters used in the mass and energy calculation are listed in Additional file 1: Table S4. The power demand of the enzyme solution pump was considered insignificant, whereas the pumps linked to the evaporator and rotary dryer (where included in the process) were incorporated in the power consumption estimate. A conceptual level estimate for fixed capital investment (FCI) costs was calculated using the chemical engineers’ factorial method. A Lang factor of 2.63 was applied based on purchased equipment costs (Towler and Sinnoit 2008). Total capital investment (TCI) was estimated by adding working capital to FCI. The TCI was fixed at 15% of the FCI.

Purchase cost estimates for major equipment were based on the Aspen Process Economic Analyzer, in-house knowledge and other sources (Towler and Sinnoit 2008; Coleman 2015; Koehorst 2020) and were scaled from the reference equipment cost using the exponential scaling equation (Eq. 1) if the reference equipment size was not equal to the designed process unit size (Peters et al. 2003). Here, cost scaling factors (S_i_) were derived from the mass balances. The reference costs (C_0_), reference scaling factors (S_0_) and cost regression indexes (exponent k) were then used to estimate the purchase cost (C_i_).

1 $${C}_{i}={C}_{0}{\left(\frac{{S}_{i}}{{S}_{0}}\right)}^{k}$$

A regression index of 0.6 was used when no better knowledge was available (Towler and Sinnoit 2008). Equipment costs were inflated to the value in 2019 euros using the Chemical Engineering magazine’s Plant Cost Index (Jenkins 2020). If necessary, we used the average currency exchange rate of 2019, where €1 = US$ 1.12 (Statista 2020).

Variable operating costs and revenues were calculated from the mass and energy balances and unit costs of raw material, chemicals, and utilities, which were based on publicly available cost data, in-house knowledge or data from project partners and collaborating companies. The price of by-product was set 60% lower than the price of the raw material because the quality of the solid residue after enzymatic treatment is yet unclear.

Fixed operational costs included labor and maintenance. Labor costs were estimated by assuming two operators per shift over five shifts, and included overheads. For scenario 1 (11 months operation), we assumed a total of 10 person-years. For scenarios 3 and 4, we assumed a labor requirement of 6 months per year, equating to 6 person-years. Annual maintenance and materials costs were assumed to be 2% of FCI, excluding chemicals used for cleaning-in-place (Peters et al. 2003).

To compare several concepts and scenarios, and to identify the most significant cost factors, the production cost was calculated (€/kg extract DM) using Eq. 2.

2 $$Production \, cost =\frac{Operational \, costs + annuity \, of \, capital \, expenditure - By-product \, revenues}{Annual \, production}$$

Taxes, interest, depreciation and amortization were excluded. The economic and financial parameters and process operation strategy are summarized in Additional file 1: Table S5.

## Results

### Composition of the chicory samples

The nutrient composition of the three chicory samples is shown in Additional file 1: Table S1. The samples differed mainly in their protein, fiber and carbohydrate levels. Total carbohydrate levels were lowest in sample #3 as expected due to the removal of inulin, and were eight-fold lower compared to the unprocessed sample #2 (Additional file 1: Table S6). The high fibre content of sample #3 is likely related to increased content of the insoluble dietary fibres during processing, as has been described earlier (Rodríguez et al. 2006; Twarogowska et al. 2020). Typical chicory STLs and polyphenols were also largely depleted during inulin extraction, as reflected by the low amounts in sample #3.

### Enzymatic treatment and antimicrobial activity

The antimicrobial activity of the chicory extracts was tested against four microbial strains. Sample #3 was also treated with different single enzymes and combinations to determine their effect on antimicrobial activity and the release of sugars (Additional file 1: Table S7). Selected enzymes were initially tested at 50 mg/g (dosing according to protein content) and the antimicrobial activity of two sample concentrations was assessed against *S. aureus*. Clear growth inhibition was observed with several enzyme treatments, but we observed independent antimicrobial activity caused by the enzyme preparations alone. We therefore reduced the enzyme dose to 5 mg/g protein.

The largest quantities of hydrolysis products (measured as reducing sugars) were released by enzymes that act on hemicellulose (xylan) and pectin, whereas glucanolytic enzymes (cellulase and β-glucosidase), esterases and inulinases showed minimal hydrolytic activity (Table 1). The combination of pectinase, xylanase and β-glucosidase inhibited the growth of *S. aureus* most effectively (Table 1). However, β-glucosidase treatment alone had no inhibitory effect so we also tested the combination of pectinase and xylanase without β-glucosidase. As anticipated, omitting β-glucosidase from the cocktail did not reduce the inhibitory effect of sample #3, confirming that pectinase and xylanase together are sufficient for strong inhibition (Table 2). All untreated chicory samples inhibited *S. aureus* to various degrees (Table 2). Pectinase plus xylanase resulted in very strong growth inhibition with samples #1 and #3, but the effect was less pronounced with sample #2. However, the treatment of sample #2 with a cocktail of pectinase, xylanase and β-glucosidase resulted in strong inhibition.

**Table 1:** Chicory sample #3 treated with individual enzymes or mixtures (5 mg/g protein) were assessed for total reducing sugars (DNS) and antimicrobial activity against *S. aureus*

Enzyme treatment	Release of hydrolysis products as reducing sugars^a^		Antimicrobial activity against *S. aureus* E-70045
	(mg/mL)	(% of dry matter)	
Inulinase	6.5	13.0	+
Pectinase	20.1	40.1	+ +
Cellulase	6.9	13.8	+
β-glucosidase	4.0	8.0	+
Xylanase	20.1	40.1	+ +
Esterase	2.8	5.5	+
All six enzymes	30.4	60.8	+ +
Inulinase + β-glucosidase	8.2	16.4	+
Pectinase + xylanase + β-glucosidase	28.1	56.2	+ + +
No enzyme			+

No inhibitory effect was observed with enzyme preparations in the absence of chicory extract. − no inhibitory effect; + weak inhibition; +  + moderate inhibition; +  +  + strong inhibition; +  +  +  + very strong inhibition

^a^Results were corrected by subtracting readings from the sample without treatment and the sample with enzyme only

**Table 2:** Chicory samples #1, #2 and #3 treated with individual enzymes or mixtures (5 mg/g protein) were assessed for antimicrobial activity against *S. aureus* and *P. aeruginosa*

Sample	Enzyme treatment	Release of hydrolysis products as reducing sugars^a^		Antimicrobial activity	
		(mg/mL)	(% of dry matter)	*S. aureus* E 70045	*P. aeruginosa* E-84219
#1	Pectinase + xylanase	15.9	31.7	+ + +	(+ + +)^b^
	Pectinase + xylanase + β-glucosidase	21.3	42.6	+ + +	(+ + +)^b^
	Pectinase + xylanase + β-glucosidase + inulinase	19.6	39.3	+ + +	(+ + +)^b^
	No enzyme			+ +	−
#2	Pectinase + xylanase	9.3	18.6	+ +	−
	Pectinase + xylanase + β-glucosidase	30.7	61.5	+ + +	−
	Pectinase + xylanase + β-glucosidase + inulinase	34.5	69.7	+ + +	−
	No enzyme			+ +	−
#3	Pectinase + xylanase	26.0	52.0	+ + +	+ + + +
	No enzyme			+	−

No inhibitory effect was observed with enzyme preparations in the absence of chicory extract. − no inhibitory effect; + weak inhibition; +  + moderate inhibition; +  +  + strong inhibition; +  +  +  + very strong inhibition

^a^Results were corrected by subtracting readings from the sample without treatment and the sample with enzyme only

^b^Growth inhibition at 24 h, complete growth recovery at 48 h

In addition to *S. aureus*, we tested the chicory extracts against the Gram-negative skin pathogen *P. aeruginosa* and two beneficial skin bacteria, i.e.* Lactobacillus rhamnosus* and *Streptococcus thermophilus*. Sample #3 strongly inhibited *P. aeruginosa* following treatment with pectinase and xylanase and was selected for more detailed investigation. Samples were taken during cultivation for chemical analysis and pH measurements. The pH dropped from 5.5 to 4.5 immediately after the cultures were initiated, whereas the pH of the control culture and untreated sample remained the same or rose slightly. Interestingly, the unprocessed chicory (sample #2) had no antimicrobial effect against *P. aeruginosa* (Table 2) whereas the Belgian endive (sample #1) showed an initial inhibitory effect (up to 24 h) but bacterial growth had recovered and was similar to the control cultures after 48 h. It was interesting to note, that sample #3 did not exhibit any inhibitory activity towards the tested beneficial skin microbes.

### Analysis of terpenes and polyphenols

**Fig. 2:**
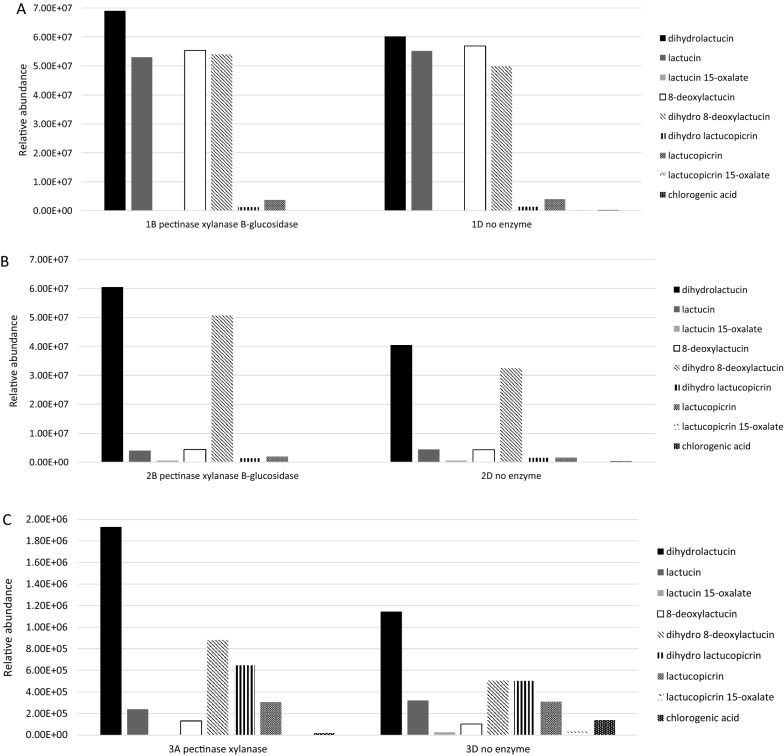
LC-Orbitrap-FTMS analysis of chicory extracts before and after the most effective enzymatic treatments. **A** Sample #1 (Belgian endive). **B** Sample #2 (industrial chicory roots, before inulin extraction). **C** Sample #3 (industrial chicory root biomass, after inulin extraction)

Enzyme-treated chicory samples with the highest antimicrobial activity (samples #1 and #2 treated with pectinase + xylanase + β-glucosidase, and sample #3 treated with pectinase + xylanase) were analyzed by LC–MS (for sesquiterpenes and polyphenols) and untargeted GC–MS (for hydrophobic compounds) compared to untreated controls. Among the phenolic compounds, we detected chlorogenic acid as well as high amounts of quinic and caffeic acids, indicating partial degradation of typical chicory hydroxycinnamoyl esters, such as isochlorogenic acid A, caftaric acid and chicoric acid (Legrand et al. 2016) (Additional file 1: Table S6). The analysis of chicory and witloof root materials revealed partial degradation of STLs during sample preparation. This was evident from low amounts of oxalated STL forms that are normally predominating in chicory (Sessa et al. 2000; Cankar et al. 2021; van Arkel et al. 2022). Additionally, the samples contained low amounts of lactucopicrin, compared to lactucin and deoxylactucin (Fig. 2). In sample #1, the main compounds were lactucin, dihydrolactucin, 8-deoxylactucin and dihydro-8-deoxylactucin (Fig. 2A) whereas only dihydrolactucin and dihydro-8-deoxylactucin were identified as major compounds in sample #2 (Fig. 2B). Enzyme treatment increased the levels of dihydrolactucin and dihydro-8-deoxylactucin in samples #1 and #2. Sample #3 contained only trace amounts of STLs and chlorogenic acid, but enzyme treatment again increased the abundance of dihydrolactucin and dihydro-8-deoxylactucin, as well as dihydrolactucopicrin (Fig. 2C). A complex mixture of compounds was detected in this sample, several with the same base peak mass, indicating structural similarity (Additional file 1: Fig. S1). Untargeted metabolomics revealed 113 metabolites overrepresented in sample #3 following treatment with pectinase + xylanase. The 10 major metabolites were analyzed by MS/MS, revealing two peaks eluting at 12.64 and 15.90 min representing sesquiterpene lactone degradation products, six nitro-alkanes, and two unidentified compounds (Additional file 1: Fig. S1 and Table S8).

The total ion gas chromatograms of the samples did not reveal any differences between the enzymatically treated and untreated samples. Samples #3A and #3D were further screened for typical masses representing monoterpenes, sesquiterpenes and triterpenes, but no such compounds were detected.

### Cosmetic formula challenge test

**Fig. 3:**
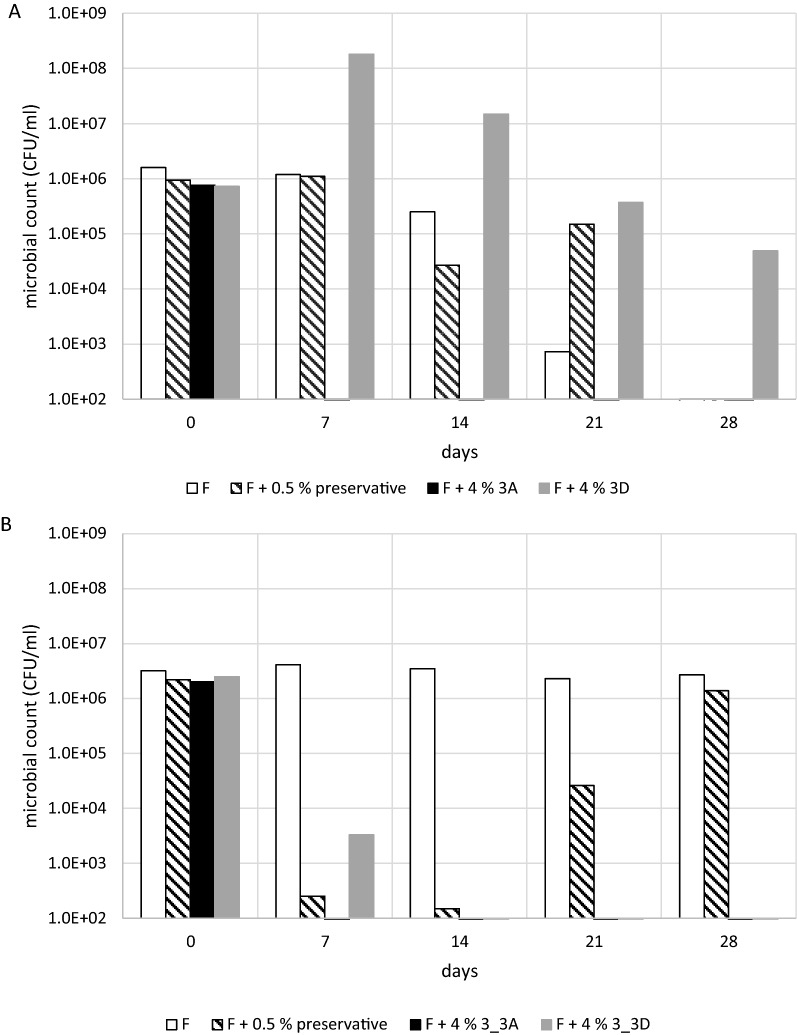
Microbial challenge test in a standard cosmetic cream formula. Samples were taken weekly for 4 weeks and incubated for 72 h with **A** *S. aureus* or **B** *P. aeruginosa* before serial dilution and colony counting. F = the basic cosmetic formula without extracts. F + 0.5% preservative = positive control. F + 4% 3A = enzyme-treated chicory extract. F + 4% 3D = untreated chicory extract

When chicory extracts were mixed into a standard cosmetic cream formula to replace a synthetic preservative, we observed a significant decline in the number of skin-related bacteria (*S. aureus* and *P. aeruginosa*) in challenge tests during the 1-month follow-up (Fig. 3).

The enzyme-treated chicory extract (sample #3A) showed strong effects after only 7 days, with the formula containing this sample strongly reducing the microbial load of both *S. aureus* (Fig. 3A) and *P. aeruginosa* (Fig. 3B). The standard 0.5% commercial preservative was also able to inhibit the growth of *S. aureus*, but only after 28 days, and of *P. aeruginosa*, but only at the beginning of the test, with the bacteria recovering toward the end. The untreated chicory extract (sample #3D) did not inhibit the growth of *S. aureus* during the 1-month follow-up (and may provide the bacteria with nutrition, including carbohydrates) but it did inhibit the growth of *P. aeruginosa*.

### Techno-economic analysis

A conceptual level techno-economic analysis was conducted to evaluate the feasibility of a process to manufacture enzymatically treated chicory extract as an antimicrobial ingredient. Pressed and dried chicory pulp is typically available all year (~ 90,000 tons per year in Europe), whereas fresh pulp is available for only ~ 5 months per year. We set our baseline case, defined as scenario 1, to 1500 tons of dry pulp with a dry matter (DM) content of 90%, which is enzymatically processed to produce an antimicrobial extract (Fig. 1). Enzymatic treatment is a batch extraction process lasting 20 h, indicating an overall processing time (including filling, extraction, inactivation, discharging and cleaning) of 24 h. The batch process starts with filling the reactor with chicory pulp, adding water and enzymes, and slow agitation. Following extraction, the enzymes are inactivated by heating and the reactor is discharged. The solid fraction and liquid extract are separated by centrifugation and collected in storage tanks to allow reactor cleaning before the next batch. The subsequent process steps are continuous to reduce the capacity of the equipment and thus the investment costs. The dilute extract is concentrated by a mechanical vapor recompression (MVR) evaporation system while the solid fraction is dried in a rotary dryer.

We considered three additional scenarios (Additional file 1: Table S3). In scenario 2, the extract is not concentrated (concentration may not be necessary for skin creams), thus reducing equipment and energy costs, but possibly increasing transportation costs and limiting the shelf life of the product. In scenario 3, we used fresh pulp with a DM content of 22%, which reduces raw material costs but also limits the annual operation to ~ 5 months based on raw material availability, thus increasing the share of capital expenditure (CAPEX). In scenario 4, enzymatic treatment is integrated with the inulin plant: the input is fresh pulp and the solid residue after enzymatic treatment is returned to the inulin plant’s pulp dryer, with 60% decrease in the DM content.

The overall mass balance was calculated based on process parameters defined in Additional file 1: Table S9. Electricity consumption in scenario 1 was 127 kW, with a heat demand 501 kW. The main mass flow rates and energy consumption in different scenarios are compared in Table 3. The distribution of electricity consumption is set out in Additional file 1: Fig. S2.

**Table 3:** Comparison of four techno-economic scenarios, all assuming the same input of raw material dry matter (1500 ton/year) and the same annual production rate of extract dry matter (1163 ton/year) and animal feed dry matter (607 ton/year)

		Scenario 1	Scenario 2	Scenario 3	Scenario 4
Annual availability	h/year	7884	7884	3592	3592
Main material streams					
Input					
Chicory pulp	kg/h	211	211	1898	1898
• Dry matter content	wt%	90	90	22	22
Output					
Extract	kg/h	295	3882	623	623
• Dry matter content	wt%	50	4	50	50
Animal feed	kg/h	86	86	188	326
• Dry matter content	wt%	90	90	90	52
Energy demand					
Electricity demand total	MWh/t_raw material(dry)_	0.67	0.38	0.67	0.66
Heat demand total	MWh/t_raw material(dry)_	2.63	2.63	2.63	2.26

**Fig. 4:**
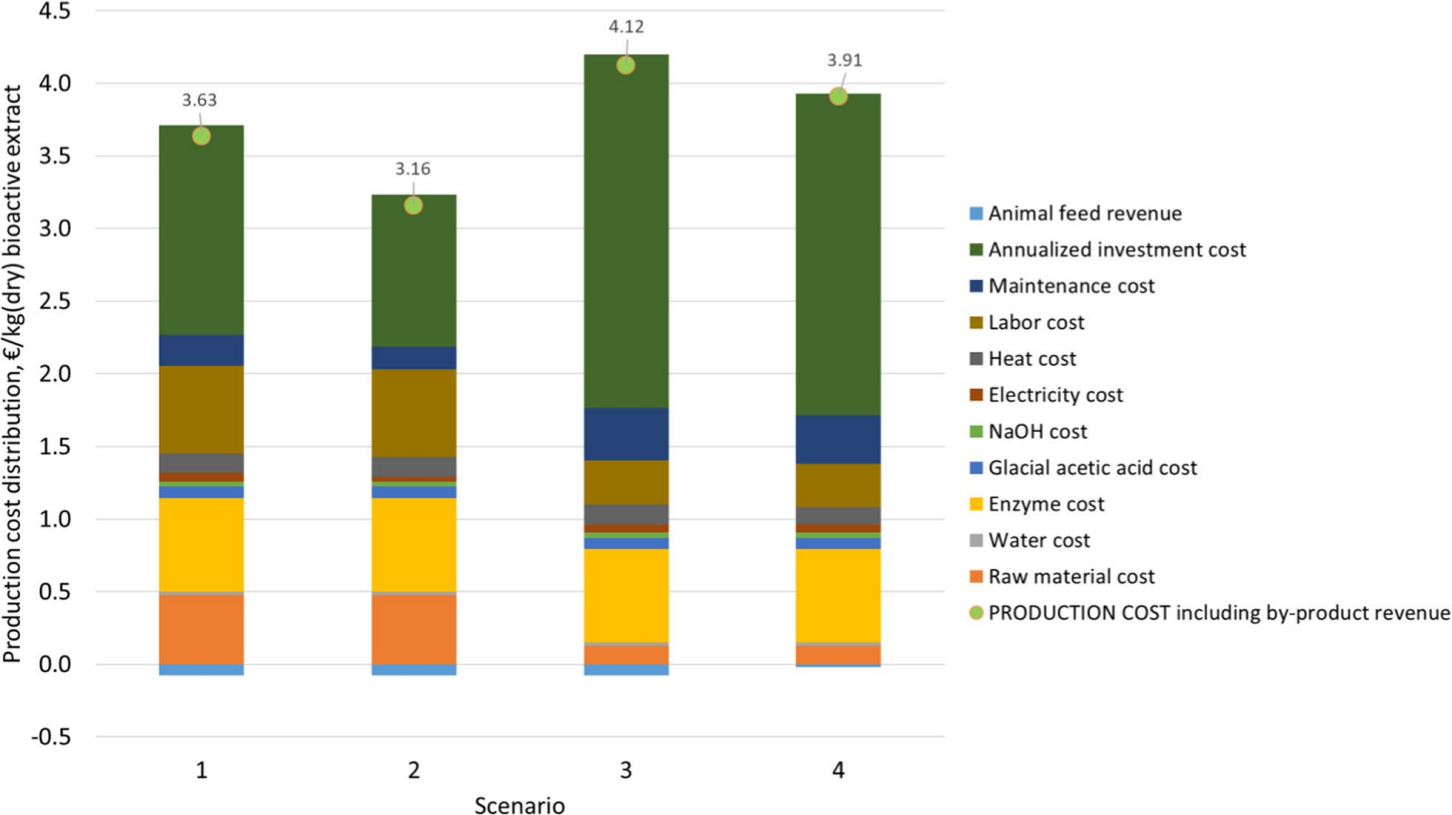
Production cost distribution in four techno-economic scenarios

We applied a Lang factor of 2.63 for the FCI costs. We considered a value of 2.0, which was proposed for a modern food processing plant with similar equipment and operating conditions (Marouli and Maroulis 2005). However, we adopted the value of 2.63 proposed by Peters et al. (2003), which is based on the value of 1.83 proposed for a similar plant (Koehorst 2020) with an increment to account for piping and buildings. The overall investment cost calculated for scenario 1 was €12.4 million for the FCI and €14.3 million for the TCI. The most expensive equipment items were the reactors and the MVR evaporator package. The number of enzymatic reactors for scenario 1 was optimized by calculating the total production cost for different numbers of reactors and choosing the number with the lowest production costs (three reactors). The same number of reactors was used in all scenarios. Only one train was considered for the reactor feeding system and downstream processing. The estimated total production cost in scenario 1 was €4.2 million/year, corresponding to €3.63/kg dry extracted compound (100% DM), although the extract contains 50% water in scenarios 1, 3 and 4, and 96% water in scenario 2. The cost distribution of all scenarios is compared in Fig. 4. The major cost factor in all scenarios is the annualized investment cost, with other significant factors including labor, enzymes and raw materials (chicory pulp). The high share of investment and labor costs reflects the relatively small capacity of the plant and indicates that there is potential for cost reduction at larger scales.

A sensitivity analysis of the production costs in scenario 1 using the key cost factors as input parameters showed that the investment and enzyme costs were the predominant factors (Additional file 1: Fig. S3). Variations in raw material and labor costs did not have a substantial effect, and the impact of the costs of energy and the by-product animal feed price were even lower. A specific sensitivity analysis of plant capacity showed that increasing the capacity influences the production cost and its distribution (Additional file 1: Fig. S4). When increasing the capacity fourfold, the number of persons in the shift increased to three, but when increasing the capacity tenfold, the number of persons in the shift increased only to four. Increasing the production capacity therefore significantly reduced the share of the fixed operating costs and the annualized investment cost, while increasing the share of enzyme and raw material costs.

## Discussion

The control of microbial growth in cosmetic products is necessary to ensure quality and safety. Microbial contamination in cosmetic products can lead to changes in the structure that are perceived as differences in viscosity, color and odor. However, ~ 6% of the population is sensitized to cosmetic ingredients, especially preservatives and fragrances (Canavez et al. 2021). Preservatives may cause skin irritation, erythema, contact allergy, contact sensitization, and contact dermatitis (Halla et al. 2018). In addition, legislation related to several currently used preservatives in cosmetics is restricting or even prohibiting their use in the future, thus the industry is actively seeking new natural ingredients for their replacement.

Application of hydrolyzing enzymes has been shown to be a viable way to treat cellulosic biomass e.g. when developing renewable energy sources or for single cell protein production (Wikberg et al. 2017; Pihlajaniemi et al. 2020). Enzymes have also been used to liberate the bioactive components from plant-based cell matrix (Hammed et al. 2013). In this study, we observed that highest amounts of hydrolysis products were released from chicory biomass by enzymes acting on hemicellulose and pectin. Concurrently, the highest antimicrobial activity against typical skin-related pathogen *S. aureus* was observed with these samples. Given that the observed antimicrobial effects were unlikely to be caused by released sugars, we assume that the enzymes also release more complex organic molecules with antimicrobial properties. The underlying mechanisms are unknown, but may reflect the degradation of the cell wall substrate (triggering the release of bioactive compounds) or undiscovered minor side activities that generate active components from chicory material by direct enzymatic action. The application of cell wall-degrading enzymes was also shown to increase the antibacterial activity of bilberry side streams (Puupponen-Pimiä et al. 2008). In addition to *S. aureus*, the same sample extract showed a strong inhibition of *P. aeruginosa*, an opportunistic human pathogen that usually causes difficult-to-treat infections for humans. Since strong activity towards this Gram-negative bacterium was observed, the potential inhibitory action towards beneficial skin bacteria, i.e.* Lactobacillus rhamnosus* and *Streptococcus thermophilus* was assessed. Remarkably, no inhibitory activity was observed. This type of selectivity is very promising when considering various applications for human use, with balanced microflora being crucial for healthy skin. Recently, we showed similar results with hydrothermal extracts of *Rubus* berries (Puupponen-Pimiä et al. 2021). The reason for low or negligible effect on growth is yet unknown, however lactic acid bacteria for example have several mechanisms to protect themselves from oxidative stress (Feng and Wang 2020) and it can be postulated that they might benefit from certain components in the sample material. In addition, they were also shown to be able to metabolize phenolic compounds in plant materials (Filannino et al. 2015). Taken together, the possibility that waste fraction of chicory industrial process could be upgraded into valuable natural cosmetic preservative is fascinating, targeting both the acute needs of cosmetic industry but also exploiting chicory as a renewable source and contributing to circular bioeconomy-based concepts. It is also interesting to note that chicory root extracts were recently clinically shown to possess a skin barrier function and vitamin D-like activity when applied topically (Maia Campos et al. 2017).

Chicory roots typically contain lactucin, lactucopicrin and 8-deoxylactucin in their oxalated forms (Sessa et al. 2000). However, the enzyme-treated chicory samples did not contain oxalated STLs, which were previously shown to be unstable during extraction (Sessa et al. 2000), indicating a partial degradation of STLs during processing. In order to understand the chemical compound(s) responsible of the strong antimicrobial activity, the pectinase + xylanase treated sample extract #3 was subjected to targeted and non-targeted chemical profiling. While GC–MS analysis did not reveal the presence of any compounds that could explain the high bioactivity, LC–MS analysis indicated the absence of typical chicory sesquiterpene and phenolic compounds but revealed a mixture of degradation products that may partially originate from chicory (STL degradation products) or inulin processing. Therefore, we concluded that the observed bioactivity was not caused by phenolic compounds or STLs and must be associated with other constituents of the extract.

Techno-economic assessment formed four scenarios showing the contributions of e.g. the moisture content of raw material and final extract concentration. The extract concentration should be carefully assessed because it has a significant effect on costs, particularly the TCI. Scenario 2 achieved the lowest production costs by omitting the evaporation system, resulting in a dilute extract (3% DM). Scenarios 3 and 4, in which the operation lasts only 5 months per year, resulted in higher total production costs than all-year operation. This means that the low raw material costs do not balance the higher investment costs at the annual capacity considered in this model, even assuming that no additional operators per shift are needed due to the doubling in capacity. The economic analysis clearly shows that because the capacity of the plant is relatively small, the most cost-effective strategy is all-year operation rather than restricting operations by the availability of inexpensive raw material. When comparing scenarios 3 and 4, integration with the inulin plant improves the economic feasibility because a rotary dryer is not required, even though the by-product price falls from €145 to €40 per ton of dry biomass. A cost-effective solution, which was not included in the scenarios, is the use of wet chicory pulp when available, and dried chicory pulp at other times, which would reduce raw material costs by more than 30%.

The profitability of the concept depends strongly on the price achieved for the bioactive extract. An average price of €130/kg was calculated for bioactive substances with multiple functions for use as functional foods and dietary supplements, based on the results of a business-to-business survey (Stern et al. 2015). In contrast, prices for basic synthetic preservatives (€1.4–4.0/kg) and natural antimicrobials (€4.2–48.2/kg) were calculated for the food industry (Davidson et al. 2020). The product described herein is intended as an antimicrobial preservative for cosmetic or personal care applications, to prevent microbial growth, and can be described as a natural antimicrobial. Based also on our in-house knowledge, the market price range is €10–100/kg dry extract. Assuming the lowest value in this range, the profitability is €7.4 million/year (assuming a production cost of €6.4/kg extract dry). The challenge will be market penetration, but current trends favor nature-derived cosmetics. The raw material for the process (dried chicory pulp after inulin extraction) is currently used as animal feed (price range = €270–400 per ton). The production of antimicrobial extracts would significantly increase the value of the by-product. The higher uncertainties in the economic assessment reflect the cost of enzymes and capital investment and represent the highest shares of overall production costs. The enzyme cost would be negotiated with the enzyme producer, and we have used a rough estimate. The uncertainty in the capital investment cost will decrease when we move from the conceptual level to a more detailed assessment.

Industrial chicory is used mainly for inulin extraction, and many bioactive compounds are lost in the discarded biomass. We found that chicory bagasse treated with hydrolytic enzymes is a source of antimicrobial extracts that inhibit human skin pathogens but not beneficial skin microbes such as lactobacilli. Activity was retained during a challenge test with a cosmetic formula, showing that the chicory extract is suitable for cosmetic and/or personal care products. The concept was also profitable based on an assumed price of €10/kg dry extract. This inexpensive and underutilized industrial chicory waste stream is a promising source of value-added bioactive ingredients.

## Supplementary Information

**Additional file 1: Table S1.** Composition of the chicory samples. **Table S2.** Composition of standard cream formula. **Table S3.** Scenarios considered.** Table S4**. Applied process parameters for mass and energy calculation based on experimental work and literature.** Table S5**. Applied economic and financial parameters and process operation strategy (location the Netherlands).** Table S6.** Amounts of selected carbohydrates, phenolic compounds and sesquiterpene lactones in the three chicory samples. Dry matter was measured by weighing. **Table S7**. Activity profiles and protein content (mg/ml) of used enzymes. Activities are expressed as nkat/ml, except cellulase activity as FPU/ml, and assayed in pH 5, except inulinase in pH 6.** Table S8.** MSn results per selected peak of LC-Orbitrap-FTMS chromatogram of sample 3A (pectinase + xylanase) (see Fig. S1): The retention time, the selected mass for fragmentation, the characteristic mass fragments and the tentative peak identification.** Table S9.** Overall mass balance of the process (baseline, scenario 1).** Fig. S1.** LC-Orbitrap-FTMS chromatogram of samples 3A (pectibase + xylanase) and 3D (no enzyme). Chromatograms are shown to scale. The peaks from the enzyme treated sample 3D that were selected for MSMS analysis are indicated by corresponding numbers.** Fig. S2.** Electricity demand in different scenarios.** Fig. S3.** The sensitivity of production cost to the selected parameters in techno-economic scenario 1. The sensitivity assessment shows the effect of one changed parameter to the production cost. The assessment was done to one parameter at a time. The lower and upper bounds of a parameter are presented in the y-axis, left the more competitive, then the base case and the less competitive. The base case is in the figure the point between the green bar and the red bar, while the green bar shows the potential up to the more beneficial value of the parameter, and the red bar the result to the less beneficial value of the parameter. **Fig. S4.** The sensitivity of production cost to capacity (scenario 1). The analysis shows the decrease of the production cost when increasing the production capacity, from the baseline (1670 t/a or 5% of the available raw material at a plant) to either 6670 t/a (20%) or 16670 t/a (50%) of the available raw material.

## Data Availability

All data generated or analysed during this study are included in this published article (and its supplementary information files).
