# Supplementary material for: Enzyme-treated chicory for cosmetics: application assessment and techno-economic analysis
Source: AMB Express. 2022 Dec 6;12:152. doi: 10.1186/s13568-022-01494-8 (PMC9727056; doi:10.1186/s13568-022-01494-8)
Supplement: Supplementary file 1 — Additional file 1: Table S1. Composition of the chicory samples. Table S2. Composition of standard cream formula. Table S3. Scenarios considered. Table S4. Applied process parameters for mass and energy calculation based on experimental work and literature. Table S5. Applied economic and financial parameters and process operation strategy (location the Netherlands). Table S6. Amounts of selected carbohydrates, phenolic compounds and sesquiterpene lactones in the three chicory samples. Dry matter was measured by weighing. Table S7. Activity profiles and protein content (mg/ml) of used enzymes. Activities are expressed as nkat/ml, except cellulase activity as FPU/ml, and assayed in pH 5, except inulinase in pH 6. Table S8. MSn results per selected peak of LC-Orbitrap-FTMS chromatogram of sample 3A (pectinase + xylanase) (see Fig. S1): The retention time, the selected mass for fragmentation, the characteristic mass fragments and the tentative peak identification. Table S9. Overall mass balance of the process (baseline, scenario 1). Fig. S1. LC-Orbitrap-FTMS chromatogram of samples 3A (pectibase + xylanase) and 3D (no enzyme). Chromatograms are shown to scale. The peaks from the enzyme treated sample 3D that were selected for MSMS analysis are indicated by corresponding numbers. Fig. S2. Electricity demand in different scenarios. Fig. S3. The sensitivity of production cost to the selected parameters in techno-economic scenario 1. The sensitivity assessment shows the effect of one changed parameter to the production cost. The assessment was done to one parameter at a time. The lower and upper bounds of a parameter are presented in the y-axis, left the more competitive, then the base case and the less competitive. The base case is in the figure the point between the green bar and the red bar, while the green bar shows the potential up to the more beneficial value of the parameter, and the red bar the result to the less beneficial value of the parameter. Fig. S4. The sensi [file 13568_2022_1494_MOESM1_ESM.docx]

**JOURNAL: AMB Express**

**Manuscript title: Enzyme-treated chicory for cosmetics – application assessment and techno-economic analysis**

Suvi T. Häkkinen^1*^, Katarina Cankar^2^, Liisa Nohynek^1^, Marjut Suomalainen^1^, Jeroen van Arkel^2^, Matti Siika-Aho^1^, Anna Twarogowska^3^, Bart van Droegenbroeck^3^, Kirsi-Marja Oksman-Caldentey^1^

^1^ VTT Technical Research Centre of Finland Ltd, Tietotie 2, 02044 Espoo, Finland

^2^ Wageningen University and Research, Wageningen, Netherlands

^3^ ILVO Institute for agriculture, fisheries and food research, Melle, Belgium

*Corresponding author:

Suvi T. Häkkinen

Senior Scientist, D.Sc.(Tech.)

Industrial Biotechnology and Food Solutions

VTT Technical Research Centre of Finland Ltd

Tietotie 2, P.O.Box 1000, 02044 VTT, Finland

tel.+358 50 561 0720; fax +358 20 722 7001

e-mail: [suvi.hakkinen@vtt.fi](mailto:suvi.hakkinen@vtt.fi)

**Table S1.** Composition of the chicory samples.

|  |  | **Moisture %** | **Ash g/100g** | **Protein g/100g** | **Fat**  **g/100g** | **Sugars**  **g/100g** | **Fibres**  **g/100g** | **Total carbohydrates**  **g/100g** | **Dry matter %** |
| --- | --- | --- | --- | --- | --- | --- | --- | --- | --- |
| **#1** | Belgian endive | 87.4 | 0.99 | 0.5 | <0.2 | 5.7 | 4.5 | 6.6 | 12.6 |
| **#2** | Industrial chicory root | 76.5 | 0.97 | 1.4 | 0.2 | 3.7 | 4.9 | 16.1 | 23.5 |
| **#3** | Industrial chicory root after inulin extraction | 72.1 | 1.58 | 2.4 | 0.8 | <0.2 | 21.3 | 1.9 | 27.9 |

**Table S2.** Composition of standard cream formula.

| **Phase** | **Compound** | **INCI name** | **Amount (%)** |
| --- | --- | --- | --- |
| A | TEGO CARE 450 | Polyglyceryl-3-methylglucose distearate | 3 |
|  | CITHROL GMS 40 | Glycerol stearate | 3 |
|  | TEGO ARGANOL 1618 | Cetearyl alcohol | 3 |
|  | BERGABEST MCT-OIL 60/40 | Caprylic/capric triglyceride | 6 |
|  | TEGOSOFT OER | Oeyl erucate | 4 |
| B | WATER | Aqua | 78 |
|  | GLYCERIN | Glycerin | 3 |

**Table S3.** Scenarios considered.

|  | **Scenarios** | **Raw material** | **Operation period (months per year)** | **Extract concentration** | **Solid residue treatment** |
| --- | --- | --- | --- | --- | --- |
| 1 | Baseline | Dried pulp | 11 | Yes | Yes |
| 2 | No extract concentration | Dried pulp | 11 | No | Yes |
| 3 | Fresh pulp | Fresh pulp | 5 | Yes | Yes |
| 4 | Strong integration to inulin plant | Fresh pulp | 5 | Yes | No |

**Table S4**. Applied process parameters for mass and energy calculation based on experimental work and literature.

|  | **Unit** | **Bioactive extract** | **Reference** |
| --- | --- | --- | --- |
| **Enzymatic treatment** |  |  |  |
| Extraction temperature | ^o^C | 50 | Experimental data |
| Duration | h | 20 | Assumption based on experimental data |
| Enzyme inactivation | ^o^C | 100 | Experimental data |
| Yield, dry extract/dry biomass (kg/kg) | wt-% | 61 | Experimental data |
| Enzyme solution input per dry biomass material (kg/kg) | wt-% | 0.5 | Experimental data |
| pH | - | 5.0 | Experimental data: acetate buffer |
| Dry biomass input per water input (kg/kg) | % | 5.0 | Experimental data |
| Mixing electricity consumption | kWh/m^3^ | 0.6 | Pereira & Leib 2008 |
| **Centrifugation** |  |  |  |
| Cake dry matter content | wt-% | 50 | 40-50 % (JRC 2018, EC 2006) |
| Solids in filtrate | wt-% | 0 | Assumption |
| Electricity consumption | kWh_e_/t_solids in_ | 15 | Assumption based on Genck et al. 2008 (10 000 rpm) |
| **Evaporation (MVR)** |  |  |  |
| Product dry matter content | % | 50 | Assumption |
| Operation temperature | ^o^C | 40 | Assumption |
| Evaporated extract | % | 0 | Assumption |
| Electricity consumption per t of evaporated water | kWh/t_evaporated water_ | 15 | JRC 2018 |
| **Rotary dryer** |  |  |  |
| Product dry matter content | % | 90 | JRC 2018 (sugar beet pulp 86-92%) |
| Extract evaporated | % | 0 | Assumption |
| Thermal efficiency | % | 55 | Genskow et al. 2008 (Thermal efficiency with steam heated air) |
| Electricity consumption per kg of feed dry | kWh/kg_in,dry_ | 0.012 | Genskow et al. 2008 (Heat sensitive solid) |
| **Other equipment** |  |  |  |
| Screw conveyor electricity consumption | kWh/t_solids_ | 0.033 | Jansen & Heuning 2021 |
| Thermal efficiency for heating buffer solution/ heating of the mixture to the inactivation temperature |  | 90 |  |
| Pump efficiency  Pump’s driver efficiency | % | 75  95 | Hannula 2015 |

**Table S5**. Applied economic and financial parameters and process operation strategy (location the Netherlands).

| **Parameter** | **Unit** | **Baseline value** | **Range** | **Reference/additional information** |
| --- | --- | --- | --- | --- |
| Dried chicory pulp price (DM 90%) | €/t dry | 370 | 300-440 | Cosucra |
| Wet chicory pulp price (DM 22%) | €/t dry | 100 |  | Cosucra |
| Enzyme solution | €/t dry | 100 000 | 40 000 - 150 000 | Estimate |
| NaOH 100% | €/t dry | 500 |  | Estimate |
| Acetic acid (glacial 99%) | €/t dry | 500 |  | Estimate |
| Process water | €/m^3^_n_ | 1.0 |  | Estimate based on EEA (2013) |
| Electricity | €/MWh | 70 | 50-100 | Eurostat 2019 (2017: Medium size industry FR 73, BE 81, NL 61) |
| Heat (low pressure steam) | €/MWh | 40 | 25-55 | Estimate |
| Waste water | €/m^3^_n_ | 1.5 |  | Estimate based on EEA (2013) |
| **Product price estimation** |  |  |  |  |
| Dried solid residue | €/t_dry_ | 145 | 117 - 172 | Estimate based on raw material price and remaining energy content* |
| **Fixed costs** |  |  |  |  |
| Labour, annual cost of a person to employer | €/a | 70 000 | 60 000 -  80 000 | Estimate (includes overheads) |
| Number of persons in a shift | - | 2 |  | Estimate |
| Number of shift (continuous operation) | - | 5 |  |  |
| Annual maintenance | % of FCI | 2.0 |  | Peters et al 2004 |
| **Operational and financial parameters** |  |  |  |  |
| Annual operation time | h/a | 7884 | 3592** |  |
|  | % | 90 | 41** |  |
| Economic lifetime | a | 20 |  |  |
| Rate of return | % | 10 | 8-12 |  |

* Cost estimation is based on dried chicory pulp price and the decreased energy content left in the solid residue. Based on the extraction process, in which 61% of material is extracted, it is assumed that remaining energy content in the solid residue is 39% and thus the price is 39% of the original chicory pulp price, consequently.

** Scenarios 3 & 4

Mass and energy balances were calculated using spreadsheet software.

**Table S6.** Amounts of selected carbohydrates, phenolic compounds and sesquiterpene lactones in the three chicory samples. Dry matter was measured by weighing.

|  | **#1** | **#2** | **#3** |
| --- | --- | --- | --- |
| Stachyose (g/100 g DW) | 0.79 | 2.88 | 0.18 |
| Raffinose (g/100 g DW) | 2.29 | 5.59 | 0.23 |
| Saccharose (g/100 g DW) | 25.22 | 8.21 | 0.43 |
| Glucose (g/100 g DW) | 0.07 | 0.11 | 0.01 |
| Fructose (g/100 g DW) | 27.27 | 10.66 | 0.31 |
| 4-OH-phenylacetic acid (µg/g DW) | 471.43 | 6.67 | 0.36 |
| Caffeic acid (µg/g DW) | 134.05 | 15.01 | 2.24 |
| Quinic acid (µg/g DW) | 4948.90 | 3902.53 | 47.63 |
| Ferulic acid (µg/g DW) | 0.27 | 0.29 | 0.07 |
| Chlorogenic acid (µg/g DW) | 455.97 | 726.47 | 26.43 |
| Chicoric acid (µg/g DW) | 1.27 | 15.15 | 4.33 |
| Lactucin (µg/g DW) | >120 | 33.18 | 3.24 |
| Dihydrolactucin (µg/g DW) | >120 | >120 | 15.48 |
| Lactucopricin (µg/g DW) | 39.89 | 13.56 | 0.42 |
| Dihydrolactucopicrin (µg/g DW) | 13.01 | 8.09 | 4.68 |
| **Dry matter (%)** | **91.4** | **90.7** | **93.2** |

**Table S7**. Activity profiles and protein content (mg/ml) of used enzymes. Activities are expressed as nkat/ml, except cellulase activity as FPU/ml, and assayed in pH 5, except inulinase in pH 6.

| **Enzyme** | **Cellu-lase ^a^** | **Endoglucanase ^b^** | **β -glucosidase ^c^** | **Xylanase ^d^** | **Polygalaturonase ^e^** | **Inulinase (pH 6,0) ^f^** | **Protein content ^g^** |
| --- | --- | --- | --- | --- | --- | --- | --- |
| Inulinase (Fructozyme L) |  |  |  |  |  | 19000 | 16.6 |
| Pectinase (Pectinex Smash) |  |  |  |  | 214600 |  | 59 |
| Cellulase  (Cellic CTec2) | 135 |  |  |  |  |  | 257 |
| β-glucosidase (Novozym-188) |  | 736 | 5908 | 2968 | 2079 |  | 104 |
| Xylanase  (Depol 40) | 23 | 21660 | 646 | 21317 | 124500 |  | 105 |
| Esterase  (Depol 740L) | 1 | 1360 | 434 | 15342 |  |  | 29 |

*Bio Rad Lowry, after acetone precipitation

^a^) Ghose (1987)

^b^) IUPAC (1987)

^c^) Bailey and Nevalainen (1981)

^d^) Bailey et al. (1992)

^e^) Bailey and Pessa (1989)

^f^) Abu El-souond et al. (2014)

^g^) Lowry et al. (1951)

**Table S8.** MSn results per selected peak of LC-Orbitrap-FTMS chromatogram of sample 3A (pectinase + xylanase) (see Figure S1): The retention time, the selected mass for fragmentation, the characteristic mass fragments and the tentative peak identification.

| **Peak nr.** | **RT (min.)** | **Parent ion [M]+*(M/Z)*** | **Characteristic MS fragment(s)** | **Tentative identification** |
| --- | --- | --- | --- | --- |
| 1 | 12.64 | 505.16783 | 475.29132 + 392.28407 + 277.11816 | Sesquiterpene lactone breakdown product |
| 2 | 15.90 | 292.21175 | 264.1805 + 188.12805 + 364.23279 | Sesquiterpene lactone breakdown product |
| 3 | 18.58 | 570.33960 | 528.29279 + 381.24234 + 592.32147 | Not identified |
| 4 | 10.30 | 302.207642 | 344.25424 | Not identified |
| 5 | 9.18 | 556.33369 | 256.32331 + 542.31812 + 539.31875 | Nitrogen containing alkane |
| 6 | 9.88 | 391.28455 | 332.21786 + 207.98514 | Nitrogen containing alkane |
| 7 | 7.53 | 391.28455 | 377.18176 + 328.22292 | Nitrogen containing alkane |
| 8 | 14.31 | 391.28455 | 247.15398 + 292.21179 | Nitrogen containing alkane |
| 9 | 15.09 | 391.28455 | 263.14908 + 308.20685 + 318.19119 | Nitrogen containing alkane |
| 10 | 8.18 | 391.28455 | 304.16547 + 207.98523 + 346.19711 | Nitrogen containing alkane |

**Table S9**. Overall mass balance of the process (baseline, scenario 1).

| **Stream** | **Total flow rate (a.r.)** | **Dry matter content** | **Dry solid content** | **Flow rate (dry)** | **PP+SL** |
| --- | --- | --- | --- | --- | --- |
|  | kg/h | % | % | kg/h dry | kg/h dry |
| **IN** |  |  |  |  |  |
| Chicory pulp | 211 | 90 % | 90 % | 190 |  |
| Water | 3 785 | 0 % | 0 % | 0 |  |
| Enzyme | 1.0 | 100 % | 100 % | 1.0 |  |
| Glacial acetic acid | 23 | 100 % | 100 % | 23 |  |
| Sodium hydroxide | 11 | 100 % | 100 % | 11 |  |
| *Total* | *4 031* |  |  | *225* |  |
| **OUT** |  |  |  |  |  |
| Animal feed | 86 | 90 % | 87 % | 77 |  |
| Extract | 295 | 50 % | 50 % | 148 | 3.9 |
| Evaporated water from rotary dryer | 63 | 0 % | 0 % | 0 |  |
| Condensed water from evaporation | 3587 | 0 % | 0 % | 0 |  |
| *Total* | *4 031* |  |  | *225* | *3.9* |


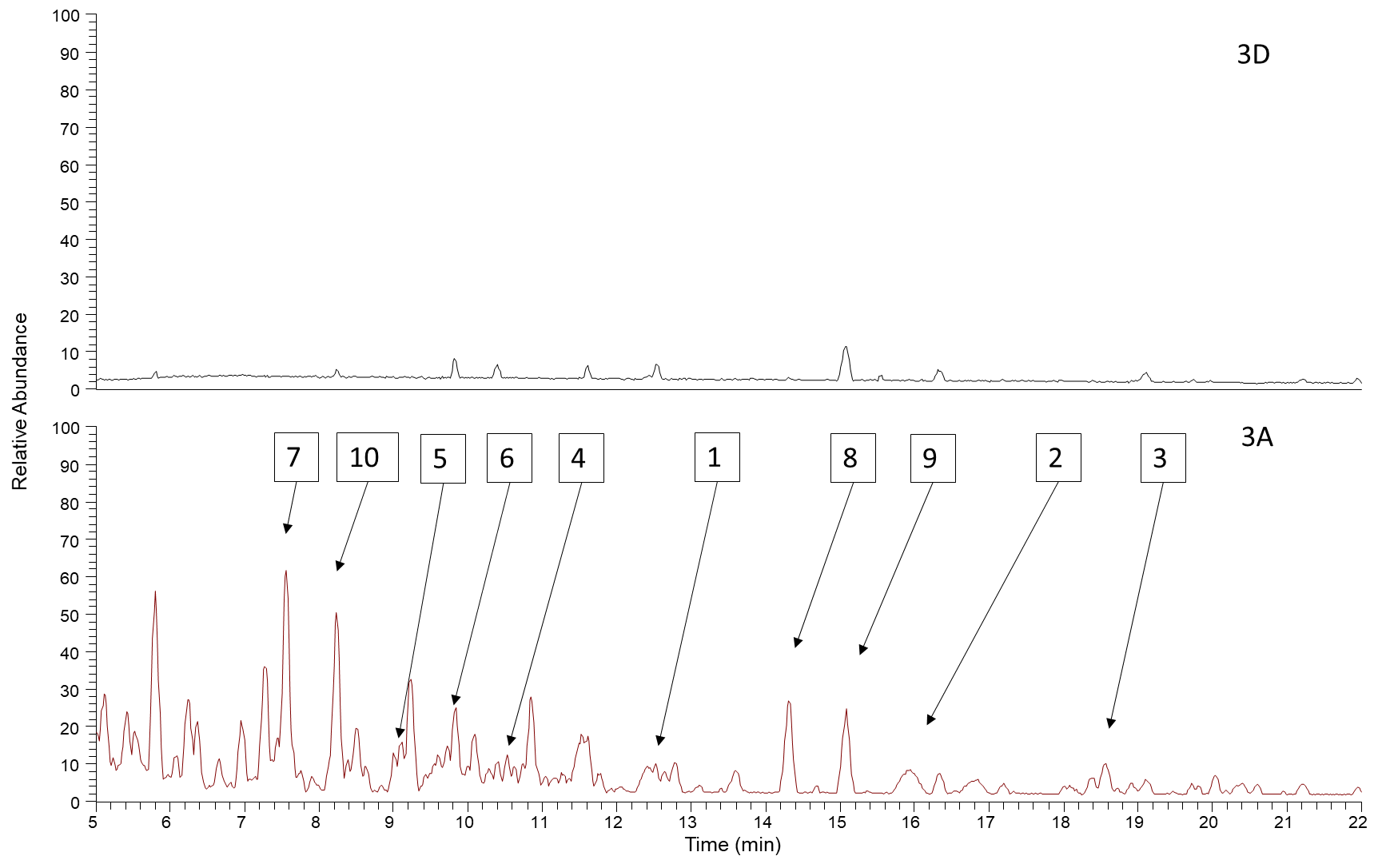


**Figure S1** LC-Orbitrap-FTMS chromatogram of samples 3A (pectibase + xylanase) and 3D (no enzyme). Chromatograms are shown to scale. The peaks from the enzyme treated sample 3D that were selected for MSMS analysis are indicated by corresponding numbers.

**Figure S2** Electricity demand in different scenarios.


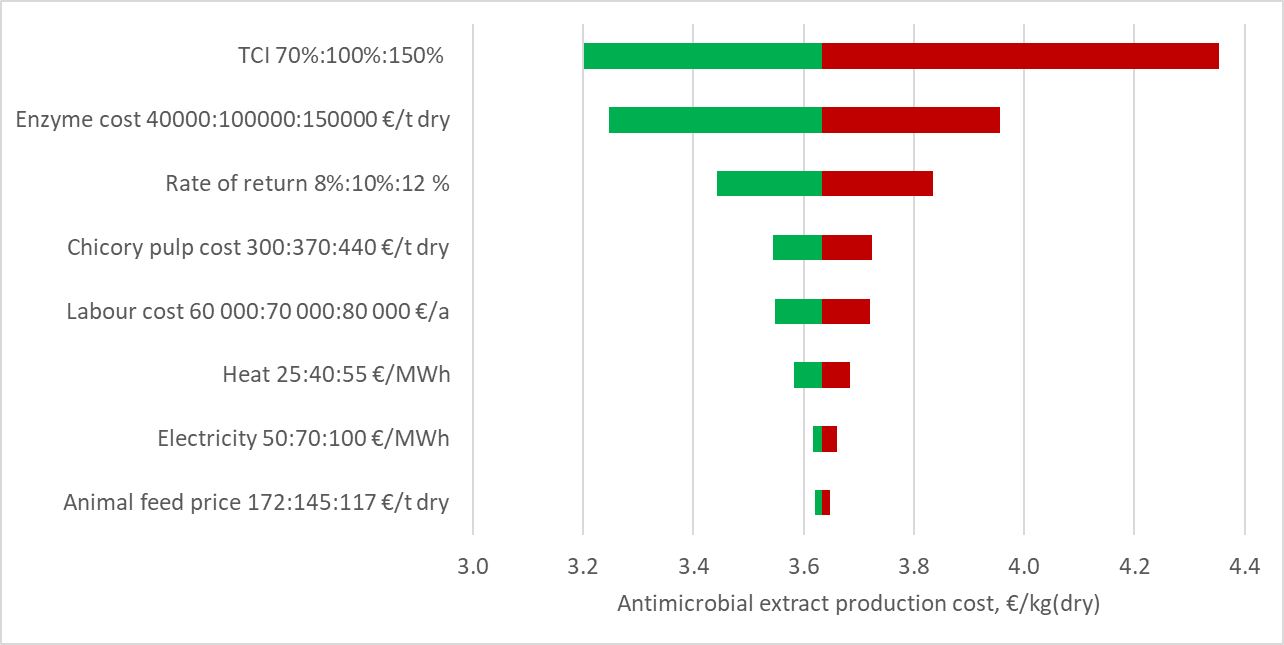


**Figure S3**. The sensitivity of production cost to the selected parameters in techno-economic scenario 1. The sensitivity assessment shows the effect of one changed parameter to the production cost. The assessment was done to one parameter at a time. The lower and upper bounds of a parameter are presented in the y-axis, left the more competitive, then the base case and the less competitive. The base case is in the figure the point between the green bar and the red bar, while the green bar shows the potential up to the more beneficial value of the parameter, and the red bar the result to the less beneficial value of the parameter.


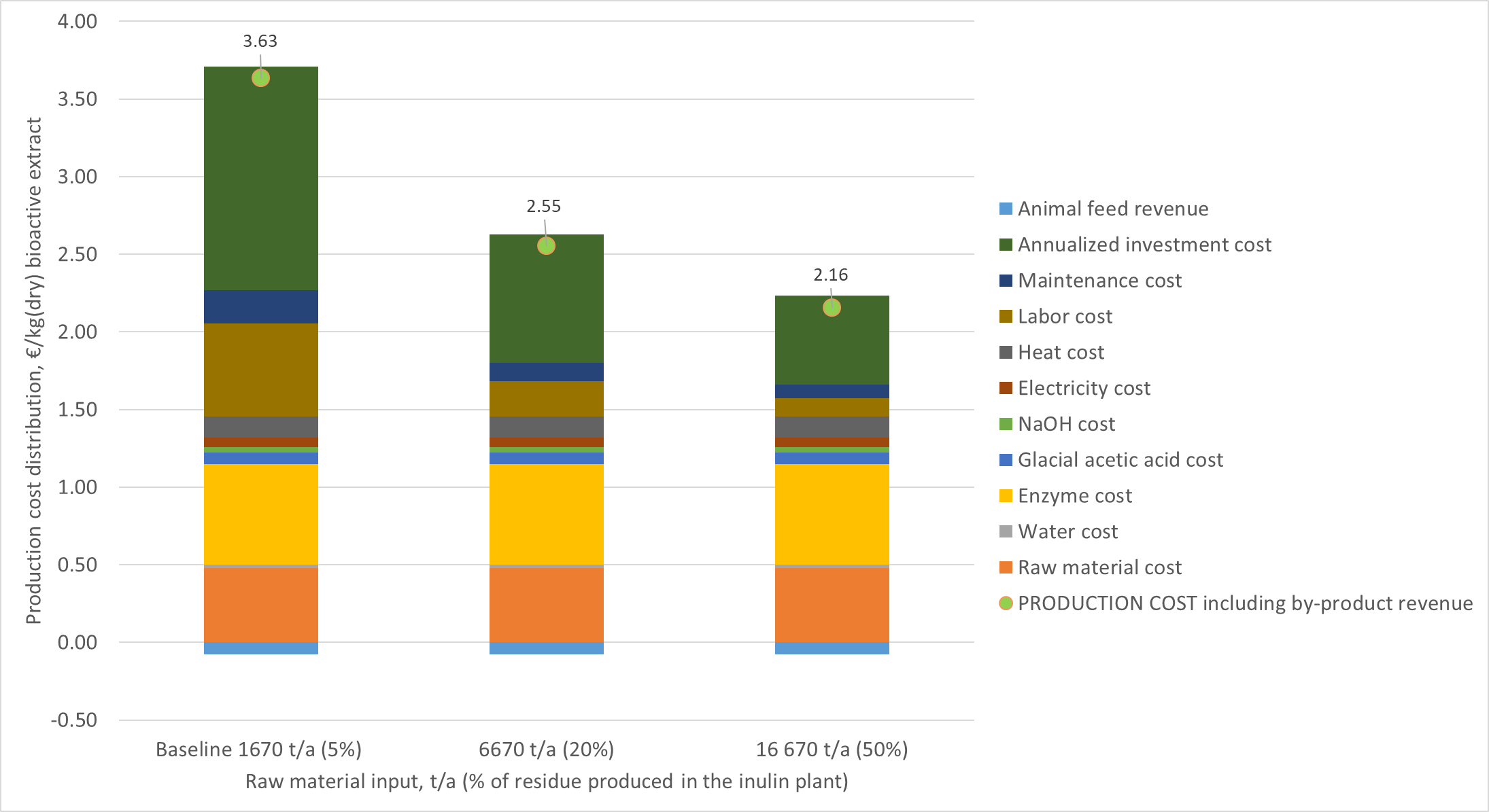


**Figure S4**. The sensitivity of production cost to capacity (scenario 1). The analysis shows the decrease of the production cost when increasing the production capacity, from the baseline (1670 t/a or 5% of the available raw material at a plant) to either 6670 t/a (20%) or 16670 t/a (50%) of the available raw material.

**Bibliography**

Abu El-souod, S. M., Mohamed, T. M., Ali, E. M. M., El-badry, M. O. & El-keiy, M. M. Partial purification of extracellular exo-inulinase from Ulocladium atrum. *J. Genet. Eng. Biotechnol.* **12**, 15–20 (2014).

Bailey, M. J. & Nevalainen, K. M. H. Induction, isolation and testing of stable Trichoderma reesei mutants with improved production of solubilizing cellulase. *Enzyme Microb. Technol.* (1981). doi:10.1016/0141-0229(81)90076-4

Bailey, M. J. & Pessa, E. Strain and process for production of polygalacturonase. *Enzyme Microb. Technol.* (1990). doi:10.1016/0141-0229(90)90098-B

Bailey, M. J., Biely, P. & Poutanen, K. Interlaboratory testing of methods for assay of xylanase activity. *J. Biotechnol.* (1992). doi:10.1016/0168-1656(92)90074-J

EC (European Commission). 2006. Best Available Techniques in the Food, Drink and Milk Industries. August 2006. Available in http://eippcb.jrc.ec.europa.eu/reference/BREF/fdm_bref_0806.pdf

EEA (European Environment Agency). 2013. Assessment of cost recovery through water pricing. EEA Technical report No 16/2013.

Eurostat. 2019. Electricity prices by type of user -Medium size industry. Available (16.4.2019) at https://ec.europa.eu/eurostat/tgm/refreshTableAction.do?tab=table&plugin=1&pcode=ten00117&language=en

Genck W.J., McGillicuddy J.K., et al. 2008. Liquid-solid operations and equipment. In Perry’s Chemical Engineers’ Handbook 8^th^ Edition, editor Don W. Green. p. 18-121.

Genskow L.R., Beimesch W.E., Hecht J.P., Kemp I.A., Langrish T., Schwartzbach C., Smith F.L. 2008. Psychometry, evaporative cooling and solids drying. In Perry’s Chemical Engineers’ Handbook 8^th^ Edition, editor Don W. Green. p. 12-77.

Ghose, T. K. Measurement of cellulase activities. *Pure Appl. Chem.* (1987). doi:10.1351/pac198759020257

Hannula I. 2015. Synthetic fuels and light olefins from biomass residues. carbon dioxide and electricity. VTT Science 107. <https://www.vttresearch.com/sites/default/files/pdf/science/2015/S107.pdf>

Jansen & Heuning. 2021. Calculation screw conveyor. Available at <https://www.jh.nl/en/berekeningsprogramma/>

JRC. 2018. Best Available Techniques in the Food, Drink and Milk Industries. Final draft version (October 2018). Available in http://eippcb.jrc.ec.europa.eu/reference/BREF/FDM/FDM_02-10-2018BW.pdf

Lowry, O. H., Rosebrough, N. J., Farr, A. L. & Randall, R. J. Protein measurement with the Folin phenol reagent. *J. Biol. Chem.* (1951). doi:10.1016/s0021-9258(19)52451-6

Pereira C.J., Leib T.M. 2008. Reactors. In Perry’s Chemical Engineers’ Handbook 8^th^ Edition, editor Don W. Green. p. 19-44.

Union, I., Pure, O. F. & Chemistry, A. International Union of Pure Commission on Biotechnology * Measurement of. *Pure Appl. Chem.* (1987). doi:10.1351/pac198759020257
